# Stochastic evolution model for international migration

**DOI:** 10.1371/journal.pone.0332886

**Published:** 2025-10-07

**Authors:** Karim Zantout, Jacob Schewe

**Affiliations:** Transformation Pathways Department, Potsdam Institute for Climate Impact Research, Potsdam, Brandenburg, Germany; Università Cattolica del Sacro Cuore Sede di Piacenza e Cremona Facoltà di Economia: Universita Cattolica del Sacro Cuore Facolta di Economia e Giurisprudenza, ITALY

## Abstract

We present a new international migration model that combines stochastic sampling techniques with dynamic accounting of flows by means of evolution equations. Migration flows are sampled from paramaterized probability distributions based on reported migration flow data that is partitioned by socio-economic covariates. This method allows for non-trivial time evolution that goes beyond extrapolation, while requiring minimal prior knowledge about the elusive processes driving migration flows. It thus combines the advantages of different existing modeling approaches. In hindcasts our model compares well with bilateral migrant stock data in many world regions and country income groups. Moreover, we observe a significant difference between the full model and its deterministic formulation, which highlights the non-Gaussian and interdependent nature of migration flow distributions and corroborates the use of a stochastic dynamic approach. Our model can be flexibly extended with additional information, e.g. regional migration policies, which are expected to further improve the agreement with data.

## Introduction

Quantitative migration models are needed to support evidence-based policy analysis and planning efforts. In addition, it can serve to counteract framings of migration that generate fear and criminalize migration or falsely attribute responsibilities [1–3]. Essentially, migration is a complex process that involves cultural, demographic, economic, geographic, historical, political, and social dimensions [4–6]. In consequence, attempts to model international human migration face intricate challenges. Moreover, these determinants are in general interdependent which makes it difficult to extract single causal relationships [7–10] as for example in the relationship between per capita GDP (GDPc) and emigration rates [11–13]. Other limitations are the varying definitions of a migrant and the differences in census across countries which make it difficult to compare migrant population data between different countries [14,15]. In addition, global bilateral migration flow data can only be estimated through approximations based on migrant population data which leads to uncertainties in the bilateral migration flows [16].

Within the migration literature we find a diverse range of models and frameworks that stem from many disciplines aiming at different research questions [4,17–19], e.g. highlighting the importance of migration networks or explaining regional differences in terms of socio-economic predictors. Following the distinction in Ref. [4] we distinguish between deterministic and stochastic migration models. The former class consists of models where migration is determined as a function of parameters and variables that are selected according to an underlying migration mechanism. Examples are linear regression models [20–25], demographic evolution equations [26–28], and macro-economic models [29–31]. These models are useful in studying certain migration channels like economic, social, and demographic migration mechanisms by incorporating specific sets of predictors, e.g. GDPc, migrant stock size, and population size, and through functional relationships between predictors and migration outcomes. On the other hand, they are limited in incorporating aspects of data quality mentioned above, such as differences in census and migrant definitions. Also, given the set of predictors and their functional relations these models are restricted to their specific migration mechanisms and may be missing significant contributions from other migration processes. For example, it was shown that gravity-type models are not able to reproduce time series of international migration [32]. Note that gravity-type model extensions can be constructed to account for time-dependent and geographical interaction effects [33,34].

In contrast, stochastic models assume that migration is a stochastic process, e.g. described as Markov process [35,36] or a posterior distribution within a Bayesian hierarchical model [37–39]. These models are better suited for acknowledging the biases in migration data and the complexity of the migration process as they work with probability distributions and rely on fewer assumptions regarding the migration process itself. In the case where migration flow data is available integrated model approaches can be used to account for data and measurement errors in combination with a stochastic migration model [40]. Nevertheless, it is not straightforward to include or extract migration mechanisms within such frameworks which contrasts with deterministic approaches where migration mechanisms are explicitly implemented in the model equations. This also means that existing stochastic models are not ideally suited for "what-if" analysis of the response of migration to changing boundary conditions. Furthermore, prediction intervals can be rather broad depending on the input data and model setup. In the specific case of Markov chain applications the Markov property poses a limitation to return migration modeling as such migration processes typically have “memory”.

In this work, we present a new international migration model based on stochastic evolution equations where we combine ideas of stochastic and deterministic approaches. This model is motivated by stochastic differential equations where the time evolution of the objects of interest is known to some degree while the underlying processes are stochastic. In the case of migration we know how to balance the different migration flows with the migrant and native population sizes but we have only limited knowledge about the migration process itself and rely therefore on a stochastic description. In order to reduce the variance of migration distributions and thus narrow prediction intervals we use partitions of the full migration data which leads to parameter-dependent migration distributions. These parameters are chosen according to demographic, economic, and social determinants of migration and hence incorporate knowledge about migration processes. As such our model can be interpreted as a stochastic version of demographic evolution approaches [26–28]. It combines demographic evolution equations with stochastic functions for migration processes to properly account for uncertainties within the migration data and complexity of migration processes. The parameterization of the migration distribution is a central difference to purely data-driven stochastic approaches such as Bayesian models [37,38,41] which allows to implicitly incorporate knowledge about migration processes or test migration theories. In this first setup of the novel approach we purely rely on a global parameterization to show the strengths of stochastic evolution modeling even down to regional benchmarks and present properties of the new framework.

We find that our stochastic evolution model can be successfully calibrated with bilateral migration data and yields parameterized migration probability distributions that are consistent with existing migration theories. Our simulated migrant population time series closely follow population estimates in many world regions and country income groups even though the model parameters are purely global, i.e. the model does not use regional or local parameterizations. For bilateral migrant stocks we investigated the top six cases where our stochastic model shows the largest deviations with reference migrant stocks. In the majority of these cases we still found that the reference migrant stocks lie within the 95% prediction interval of the stochastic model predictions. The agreement with migration data estimates declines when the full stochastic evolution model is replaced by its deterministic version where all stochastic samplings are replaced by the median value of the prior distributions. This indicates that the non-Gaussian structure and interdependence of migration distributions are key features of migration processes. We also find that diaspora ties are an orders of magnitudes stronger destination predictor than the wealth ratio between destination and origin. Therefore, our new approach provides a framework for quantitative migration models without strong assumptions on the migration mechanisms. Simultaneously, it allows to conservatively implement or test migration theories through data partitioning. Moreover, differences to time series estimates indicate the direction of potential improvements on our global parameters such as regional migration policies. These regional calibrations would turn our global parameters into regional parameters implementing local contexts of migration processes. Our approach is especially useful for future scenario building and projections as the stochastic setup allows for a flexible parameterization of the prior distributions.

## Materials and methods

### Methods

#### Evolution equations.

Within a country we distinguish between the native population *P* and migrant population *D* which are distinguished through country of birth. This choice is due to the majority of countries accounting for migrants based on a country of birth definition [42]. These groups are connected via emigration flows *M* from an origin population towards a migrant population, return migration flows *R* from a migrant population back to the origin country and birth flows *B* from a migrant population into the native population of the residence country. We do not account for transit migration, namely migration from a migrant population to a migrant population in a different country. The reason for this simplification is that transit migrations are in total one order of magnitude smaller than emigration and return migration [16] and that neglecting this type of migration reduces modeling assumptions.

We first describe the time evolution of the native population size in country *i* from time step *t*_*n*_ to *t*_*n* + 1_ by different migration flows, mortality, and birth. The dynamics can be described via

$$P_{i}(t_{n+1})=\;\left(P_{i}(t_{n})-\sum_{l}M_{il}(t_{n})\right)\cdot \left(1+c_{i}(t_{n})\right)+\sum_{l}\sum_{m=-\infty }^{n}\left[R_{li}(t_{n};t_{m})+B_{li}(t_{n};t_{m})\right],$$ (1)

where $P_{i}(t_{n})$ is the population size in country *i* at time $t_{n}$, $c_{i}(t_{n})$ is the natural population change rate between $t_{n}$ and $t_{n+1}$ due to birth and mortality, $M_{il}(t_{n})$ are the people emigrating from country *i* to country *l* at time step $t_{n}$, $R_{li}(t_{n};t_{m})$ are return migrants from the migrant population in country *l* that originally arrived at time step $t_{m}$ back to country *i* at time step $t_{n}$, and $B_{li}(t_{n};t_{m})$ are the people born at time step $t_{n}$ to the migrant population in country *i* that originally arrived at $t_{m}$ from country *l*. The first addend describes the population size at time $t_{n}$ reduced by emigration and propagated through birth and mortality. The remaining two terms describe return migration to *i* and birth from local migrants into the native population of country *i*. The *l*-sum in Eq (1) runs over all countries and accounts for all incoming and outgoing migration flows and births while the time summation aggregates all migrant arrival times in the past. A numerical example that explains each component in Eq (1) is presented in S2 Appendix.

Migrant fertility and mortality rates may be different to the ones from the origin country due to many factors such as adaptation, family reunification and health care availability [43–48]. For simplicity we assume the migrant population change rates to equal the ones from the origin country.

Next, we study the time evolution of the migrant population. In order to account for the time duration that people have been living as migrants in a specific country we model the migrant population with two time arguments: The first time argument is the actual time and the second one is the time of arrival in the residence country.

Taking all flows into account leads to

$$D_{i}^{k}(t_{n+1};t_{m})=\left\{ \begin{array}{cc} D_{i}^{k}(t_{n};t_{m})\cdot \left(1+c_{k}^{†}(t_{n})\right)-R_{ik}(t_{n};t_{m}) & n\geq m\\ M_{ki}(t_{m-1}) & n=m-1\\ 0 & \text{else}, \end{array}\right.$$ (2)

where the first case accounts for population changes in the migrant stock after its creation, namely due to mortality and return migration. The death rate $c_{k}^{†}(t_{n})$ applies to country *k* at time step $t_{n}$. The second case covers the creation of a migrant population through migration inflows at time $t_{m}$. Note that the migration inflows are not rescaled in terms of deaths and births as the underlying estimates already contain natural changes (see Data section and [16]). Finally, the last case is a boundary condition that ensures that no migrant population exists before its creation.

**Fig 1:**
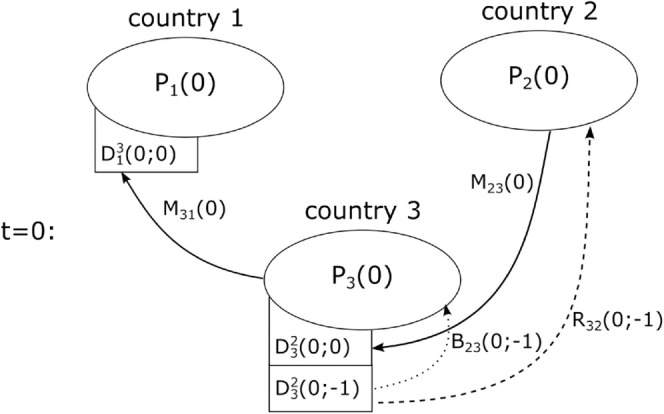
Model representation. Graphical representation of the migration model for the simple case of three countries. The arrows represent emigration (solid), return migration (dashed) and birth flows (dotted). For simplicity we have reduced the flows and migrant stocks in this representation to a minimum. This implies that we do not display all possible emigration, return migration and birth flows for readability.

A simplified graphical representation of the migration model for a simple three-country system at time *t* = 0 is shown in Fig 1. The time evolution Eqs (1) and (2) describe how the native and migrant population evolve between time steps through natural changes and migration flows. As such, they balance migration flows and respective native and migrant populations which is why we also refer to them as demographic accounting equations. Meanwhile, the complexity of the migration process is hidden in the emigration flows *M*, the return migration flows *R*, and the birth flows *B*.

#### Emigration.

We model emigration from the native population as a two-step stochastic process, where we first determine the emigration rate and then the destination countries. Both steps require calibration data such as estimated bilateral migration flows and socio-economic parameters. The details about the data sources and preprocessing are described in the Data Section.

**Fig 2:**
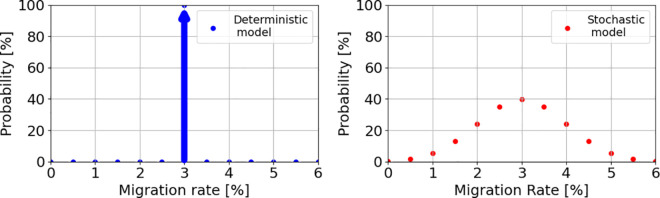
Deterministic vs stochastic model. Illustration of a deterministic (left) and stochastic (right) migration function. In the example of the main text the deterministic model produces a migration rate of 3% which corresponds to a probability distribution with a single peak at 3% and probability 100% while all other migration rates have zero probability. In the case of the stochastic model (right) the migration function produces a probability distribution where the maximum probability is 40% for a migration rate of 3% but close-by migration rates still have a non-zero probability of occurrence.

*Emigration rates.* For the reasons outlined in the Introduction we decide not to assume specific emigration mechanisms that lead to deterministic migration rates, namely explicit migration rates that result from a migration equation [4]. Instead, migration will be represented as a stochastic mechanism where migration occurs with a specific migration rate but the rates are not fixed by a set of predictor variables but rather have a certain probability depending on the predictor variables. To illustrate the difference, we assume a simple migration model with two predictor variables *GDP* and native population size *P*. If the model is deterministic, there will a function *M*(*GDP*,*P*) with arguments *GDP* and *P* that calculates the migration rate explicitly, e.g. for GDP = 10^8^USD and native population size *P* = 10^6^ we may get a migration rate $M(10^{8},10^{6})=3\%$. In a stochastic model the result of *M* is not an explicit prediction of migration rates like 3% but a probability distribution which assigns each migration rate a probability of being observed, e.g. *M*(10^8^,10^6^) could be a Gaussian probability distribution with parameters μ=3% and $\sigma^{2}=1$. While the deterministic model predicted a migration rate of 3% with probability 100% and all other migrations rates with zero probability, the stochastic model is more conservative and predicts a migration rate of 3% with probability 40% and smaller probabilities for migration rates close to 3% (see Fig 2).

**Fig 3:**
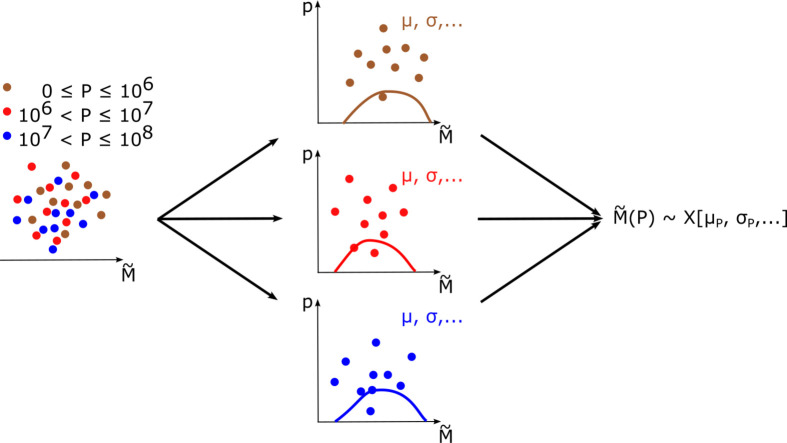
Data partitioning. Reduced illustration of the emigration rate modeling with only three labels. Given the emigration rate data (left) we label the data according to the population size in the origin country. This decomposition leads to different parameters μ,σ,… in the probability distribution function regression (middle) where the resulting probability distribution functions $p(\tilde{M})$ are shown as solid lines. These parameters can be recombined into a stochastic function $\tilde{M}(P)$ that is based on the three different probability distributions (right).

For our migration model we derive migration probability distributions from the full emigration rates data and use predictor variables to partition the data. The partitioning aims at reducing the variance of each labeled data set which consequently reduces the spread of our migration predictions. This approach implicitly incorporates prior knowledge on migration mechanisms, namely through the partitioning along selected predictor variables. On the other hand, we do not loose information that goes beyond this knowledge as partitioning does not reduce the amount of data/information but only distributes it along subsets. Specifically, we use the correlation between native population size of the origin country *P* and emigration rates $\tilde{M}$ to parameterize the emigration data with labels $P_{1},P_{2},P_{3},P_{4}$ (see Fig 3 left), namely

$$P_{1}:=[0,10^{6}],\;P_{2}:=(10^{6},10^{7}],\;P_{3}:=(10^{7},10^{8}],\;P_{4}:=(10^{8},\infty ).$$ (3)

We choose to partition the emigration rate data with respect to different orders of magnitude in population size to get enough statistics in each bin while also differentiating between smallest countries *P*_1_ and largest countries *P*_4_.

**Fig 4:**
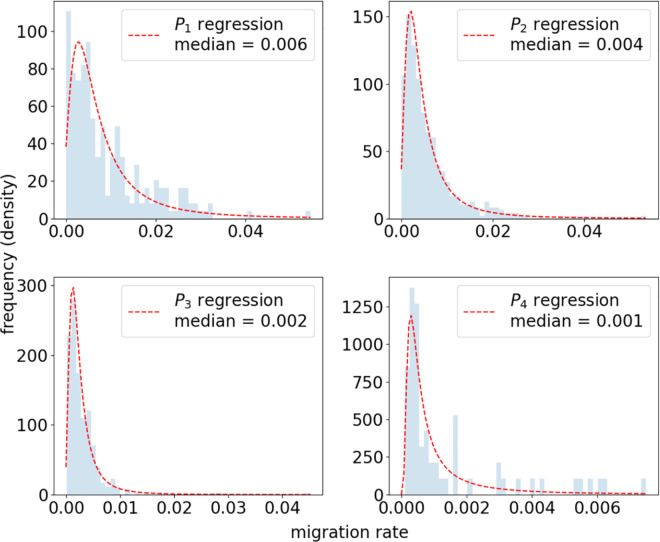
Emigration rate distributions. Emigration rate regression results (red line) for all four partitions $\left\{P_{1},P_{2},P_{3},P_{4}\right\}$ where the blue histogram represents the partitioned emigration rates data. We observe a narrowing of the distribution with increasing population size.

Our approach is motivated by the importance of the origin population size as a migration predictor [31,33] and ideas of the radiation model [49]. Within the radiation model migration flows depend solely on population sizes within different regions. The performative model essentially assumes large migration flows towards higher populated regions and small migration flows towards lower populated regions. Therefore, population size is related to migration aspirations which leads to emigration if these aspirations coincide with the necessary migration abilities. In principle, more migration determinants (e.g. legal, economic or social factors) can be included to further partition the emigration data and include additional knowledge on migration mechanisms. On the other hand, we already observe a strong differentiation in the emigration rate distributions as a function of $P_{i},\;i=1,2,3,4$, as well as limited sample sizes in the cases of very small and very large countries, namely $P_{1}$ and $P_{4}$ (see blue histograms in Fig 4). Further partitioning poses a challenge in the density estimation that is the next step in the modeling process and described in the following paragraph.

We take Jones and Faddy skew-t probability distribution functions with parameters $\left(\mu_{P_{i}}^{m},\sigma_{P_{i}}^{m},a_{P_{i}}^{m},b_{P_{i}}^{m}\right),$ i=1,2,3,4, and fit them to the respective labeled data set (see Fig 3 middle). This distribution yields the smallest errors in the regression analysis. The details of procedure which we performed to obtain the best-fitting probability distribution are described in S1 Appendix. In Fig 4 we present the resulting probability distribution functions. While the probability density functions in the cases *P*_2_ and *P*_3_ closely follow the emigration rate data we find no smooth histogram shape in the other two cases which is due to smaller sample sizes. In addition, we find a narrowing of the probability distribution when moving towards larger countries also expressed in the decrease of the median value from 0.006 to 0.001. This observation is consistent with our expectation that, in general, larger countries can offer more opportunities when it comes to education, family, work etc. and therefore exhibit smaller emigration rates.

Having obtained a probability distribution for each label we can now reconstruct a single emigration rates probability function in the following way: First, take the population size *P* of the country of interest and determine into which bin $\left\{P_{1},P_{2},P_{3},P_{4}\right\}$ it falls. For example, if 50 Mio. people live in country X the corresponding label is $P_{2}$. The correct probability distribution for country X is then $X[\mu_{P_{2}}^{m},\sigma_{P_{2}}^{m},a_{P_{2}}^{m},b_{P_{2}}^{m}]$.

Mathematically, this approach to construct a stochastic emigration rate function $\tilde{M}(P)$ amounts to combining the probability distributions through case distinction, namely

$$\tilde{M}(P)\sim X[\mu_{P^{*}}^{m},\sigma_{P^{*}}^{m},a_{P^{*}}^{m},b_{P^{*}}^{m}],$$ (4)

where P^*^ is the element in the partition $\left\{P_{1},P_{2},P_{3},P_{4}\right\}$ that contains *P* and *X* is the Jones and Faddy skew-t probability distribution (see Fig 3 right). Consequently, we make a simple step-function approximation in *P*-space.

*Emigration destination.* In the next step we determine the destination countries of the emigration flows based on the bilateral emigration share data $m_{ij}:=M_{ij}/M_{i}$, where $M_{ij}$ is the migration flow from country *i* to country *j* and $M_{i}$ is the total emigration flow from country *i*. This time our data labels are per capita GDP (GDPc) ratios $g_{ij}:=g_{i}/g_{j}$ and the relative migrant population size $d_{i}:=D_{i}/P_{i}$, where *i*,*j* are country labels and *g*_*i*_ is the GDPc in country *i*. The importance of diaspora networks for facilitating migration has its origin in reducing risks and costs of migration or support within the destination country [17,50–53]. On the other hand, the GDPc ratio contains information on economic differences between two countries and therefore is used as a proxy for migration aspirations such as labor opportunities. Thus, the partition for the emigration destination is given by

G×D, (5)

where the GDPc ratio partition *G* and relative migrant population partition *D* are defined by

$$G:=\{g_{1},\ldots ,g_{5}\},$$ (6)

$$g_{1}:=(-\infty ,10^{-1}),g_{2}:=[10^{-1},1),g_{3}:=[1,10^{1}),g_{4}:=[10^{1},\infty ),$$ (7)

$$D:=\{d_{1},\ldots ,d_{5}\},$$ (8)

$$d_{1}:=(-\infty ,10^{-7}),d_{2}:=[10^{-7},10^{-5}),d_{3}:=[10^{-5},10^{-3}),d_{4}:=[10^{-3},0.1),d_{5}:=[0.1,\infty ).$$ (9)

**Fig 5:**
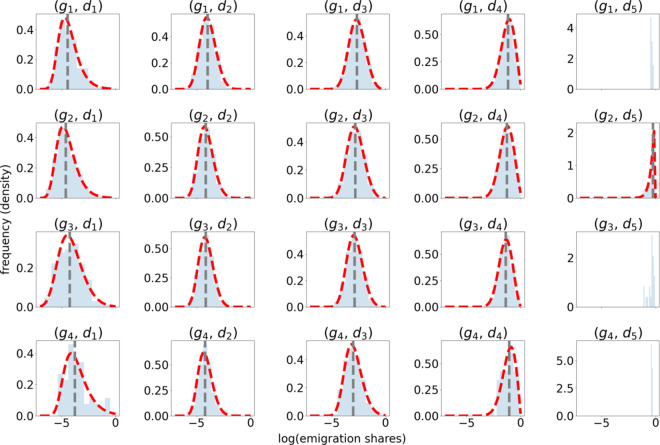
Emigration share distributions. Partitioned bilateral emigration shares data and resulting probability density functions as estimated from the regression analysis. We find that the relative migrant population size correlates with the emigration shares while the GDPc ratio has only a minor influence on the shape of the probability distribution function. The median value is marked by a dotted vertical line. In the cases where no probability distribution function line is shown we have not enough data point to perform the regression.

We again partition the bilateral emigration shares with respect to different orders of magnitude to get enough statistics within each bin and split the data into significantly different socio-economic and diaspora groups. The labeled bilateral migration share data sets are then fitted to Weibull maximum probability distribution functions (see Fig 5). The details of the regression analysis are discussed in S1 Appendix.

We observe that higher emigration shares are expected in cases where the relative migrant population size is large which is consistent with migrant network theory [17]. On the other hand, the GDPc ratio between origin and destination country has a much smaller effect on the destination country which further challenges the simplified picture of migration being reduced to economic factors such as GDP. Instead, we find that the migration destination shares can change by orders of magnitudes as a function of the existing migrant populations.

In analogy to the emigration rates we can again combine the probability distributions to construct a stochastic function for the emigration shares which can be then multiplied with the emigration rates (Eq (4)) to obtain the bilateral migration function

$$M(P,g,d)\sim \tilde{M}(P)\cdot Y[\mu_{\left(g^{*},d^{*}\right)}^{s},\sigma_{\left(g^{*},d^{*}\right)}^{s},a_{\left(g^{*},d^{*}\right)}^{s},b_{\left(g^{*},d^{*}\right)}^{s}],$$ (10)

where (g^*^,d^*^) is the element in the partition G×D where (*g*,*d*) is contained and *Y* is the Weibull maximum probability distribution. Note that in general the sampled bilateral migration flow shares do not add up to one. In these cases we perform a uniform renormalization of the emigration shares.

Finally, the bilateral migration rates *M* (Eq (10)) are multiplied with the population size of the origin country *i*, $P_{i}$, to get the bilateral migration flows $M_{ij}$, i.e.

$$M_{ij}(t_{n})=P_{i}(t_{n})\cdot M(P_{i}(t_{n}),g_{ij}(t_{n}),d_{i}(t_{n})).$$ (11)

Note that the model parameterization in Eq (11) is purely global and contains no regional effects.

#### Return migration.

Return migration describes the return of migrants from a migrant population into their country of origin. In principle, we could repeat the scheme for the emigration rates and find an adequate partition for the return migration data to obtain a parameterization of the return flow probability distribution function. As the return migration rates are already strongly centered around the median value (see S1 Figure) we will proceed without a partition of the data. Therefore, we use the approximation

$$R_{ij}(t_{n};t_{m})=D_{i}^{j}(t_{n};t_{m})\cdot R,$$ (12)

where *R* is a Student’s t distributed random variable. The details of the regression analysis and parameters of the probability distribution function are shown in S1 Appendix.

#### Birth flow.

Given that we apply a country of birth definition for migrants the birth flow *B* is calculated as

$$B_{li}(t;t_{m})=D_{i}^{l}(t_{n};t_{m})\cdot c_{l}^{*}(t_{n}),$$ (13)

where $B_{li}(t;t_{m})$ is the birth flow at time step $t_{n}$ from the migrant population that arrived at time step $t_{m}$ in country *i*, $D_{i}^{l}(t_{n};t_{m})$ is the size of the respective migrant population, and $c_{l}^{*}(t_{n})$ is the natural birth rate of country *l* at time step $t_{n}$.

### Data

The central ingredient for the migration model are the bilateral migration flows obtained from the Pseudo-Bayesian demographic accounting method [16]. These estimates typically underestimate true migration flows but are consistent with reference migrant stock data. This caveat is partly accounted for in our stochastic approach since the emigration rate distributions (see Fig 4) exhibit a heavy tail which allows for sampling higher emigration rates. As the data set contain return migration flows that are larger than 100% we simply discard them as they are less than 0.1% of the total data points. In addition, we need to rescale the 5-year return and emigration rates $m_{5year}$ to yearly rates $m_{1year}$ which is performed by the formula [54]

$$m_{1year}=1-\sqrt[{5}]{1-m_{5year}}.$$ (14)

Note that this approximation assumes homogeneous migration rates within each 5-year interval in addition to compensating emigration and return migration processes on the one-year level. Therefore, this downscaling procedure cannot be expected to match the true yearly migration rates due to concurring processes such as return migration, birth or death of migrants within the 5-year period [55,56]. There is no trivial and assumption-free way to translate migration rates between different time widths which is also known as the one-year/five-year problem [55,57,58]. Within our model we perform the same systematic error for both emigration and return rates which can partly compensate each other and we do no rely on the specific migration rates but on the distribution of the migration rates. Therefore, we make the weaker assumption that the transformation in Eq (14) conserves the scale and shape of the migration rate distributions. Note that yearly migration rates between *t* and t+1 are measured with respect to the native population at time *t*. This definition ensures consistency with our time evolution equations (Eqs 1 and 2).

The population data and natural population change rates are obtained from the United Nations World Population Prospects 2024 [59]. While natural population change rates depend on age and sex we only use total natural change rates which are aggregates of the underlying heterogeneous cohort change rates. In the case of missing population data for certain years we use linear interpolation.

The global migrant stock data from Ref. [42] contain no details on the arrival time in the residence country. This poses no problem in our modeling because simply assuming that all migrant stocks were created in the year where the simulation starts has no effect on the stocks and flows (see Methods Section). The reason is that the model approximations discard the arrival time dependency in the return and birth flows (see Eq (12) and (13)).

As for the GDP data from Ref. [60] we first collect all available PPP data and fill the missing values from MER data. For the remaining points that are missing we rely on USD data and the remaining missing values are filled with data from the closest available year. In the end we obtain demographic and economic characteristics of 192 countries which we use for the model simulations (see S5 Appendix).

## Results

All presented results were performed with a total sample size of 100.000 with the simulation initialized in 1990 and ending in 2020. The details on the sampling procedure and the convergence analysis are presented in S4 Appendix and S3 Appendix, respectively.

### Global comparison with reference data

**Fig 6:**
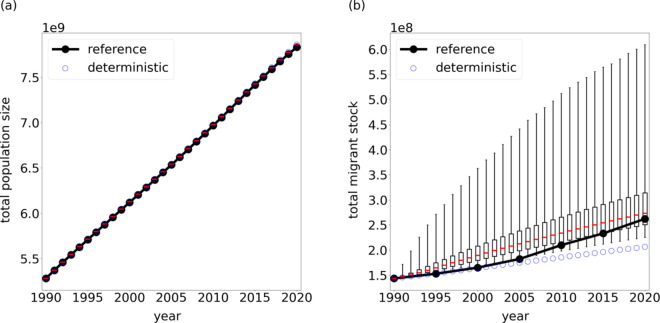
Global comparison. Comparison of the (a) total population and (b) total migrant stock between the full stochastic evolution model and the deterministic version of the model. The red bars mark the median value while the vertical boxes mark the 50% prediction interval and the whiskers denote the 95% prediction interval.

In Fig 6 we show the (a) total population size and (b) total migrant stock from data estimates (black) and from the model simulation (red).

While the red bars show the median value of each simulation year the vertical boxes and whiskers signify the 50% and 95% prediction interval respectively. In the case of the total population size we find only minor differences to the reference data that are visible towards the end of the projection interval. This strong agreement with reference population data is due to the inclusion of estimated birth and mortality rates in the native and migrant population evolution equations (Eqs (1) and (2)) highlighting the importance of flow accounting within our framework. In the case of the total migrant stock we find close agreement with the data estimates. More specifically, the observation estimates always lie within the 95% prediction interval at all times. Moreover, we observe that the 95% prediction interval is in the same order of magnitude as the total migrant stock.

In order to estimate the influence of the stochastic component of the model we derive a deterministic model version by replacing all random samples by the median value of the respective underlying distributions. We show the results for the total global population and migrant stock of the deterministic model version (blue) in Fig 6. The differences between the deterministic and the stochastic approach are most significant in the total migrant stock (Fig 6(b)). Over the full time period we find a quasi-linear increase in the total migrant in the deterministic case while this future is only visible for the median value of the full model. In general, we find that the migrant stocks lie within the 95% prediction interval at all times while the deterministic model only coincides with the migrant stock estimates for the four time steps. For the remaining time steps the deterministic results are smaller than the migrant stock estimates. We attribute the better performance to the non-Gaussian and coupled nature of the underlying probability distributions which also leads to the tendency towards larger migrant stock (cmp. Fig 4).

### Regional comparison with reference data

**Fig 7:**
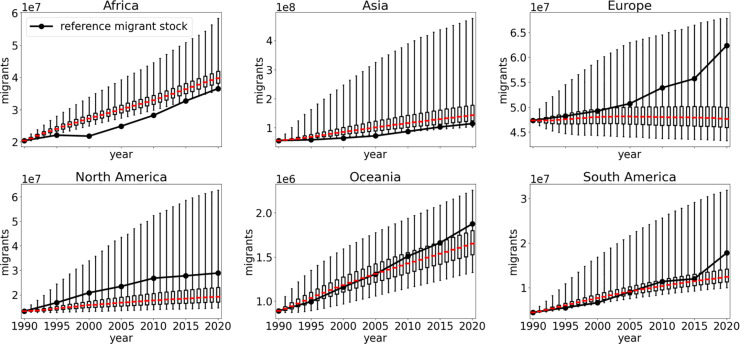
Origin region results. Migrant stocks with respect to the origin region. The red line marks the median value of the simulation while the boxes indicate the lower and upper quartile values. The whiskers mark the 95% prediction interval.

To investigate the model performance on a smaller geographical scale we present the simulated migrant stocks from different world regions in Fig 7. The mapping from countries to world regions is performed on a geographical basis as implemented in the geopandas package [61] (see S5 Appendix).

We find agreements within the 95% prediction interval for migrant stocks in all world regions except for Africa for all prediction times. In the case of Africa the lower 95% range limit is close to or partly include the reference migrant stocks. For Europe specifically, we find that the median value exhibits a stagnant migrant stock while the reference data shows an increase over time. These missing emigration flows are partly due to historic events such as the end of the Soviet Union which triggered significant changes in migration patterns within Europe [62,63].

Additionally, we find that our model overestimates the migrant population from Africa. While the increase rate of the median value is close to the reference the model is unable to reproduce the stagnation that occurred between 1995 and 2000. Considering the increasing visa restrictions within the region that were enacted in this time period and influenced the migration patterns [64] we assume such political conditions to be important aspects for improvement.

**Fig 8:**
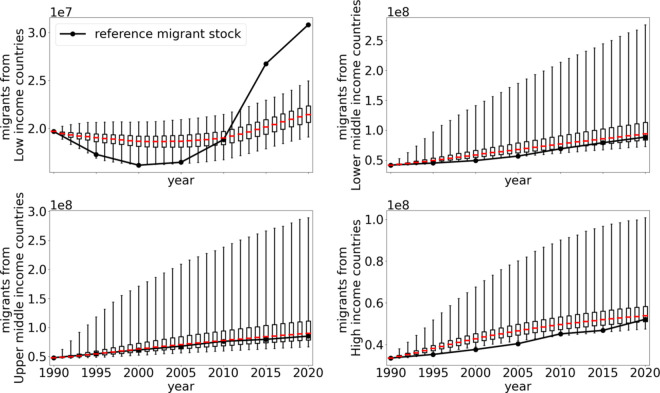
Origin income group results. Migrant stock from different origin income groups. The red line marks the median value of the simulation while the boxes indicate the lower and upper quartile. The whiskers show the 95% prediction range.

In Fig 8 we show the total migrant stock aggregated in income groups of the origin region.

The agreement with reference migrant stocks extends to all income groups expect for low income countries where our model does not predict the increase in migrant stock between 2010 and 2020. Additionally, we find that the migrant stocks in high income countries are slightly overestimated in 2000 and 2005. The reason for the strong increase in low income countries at the end of the reference interval is due to a large number of forced displacements, unprecedented in modern history, from 2010 on [65]. The respective results for destination regions and income groups are presented in S6 Appendix.

### Country-level comparison with reference data

**Fig 9:**
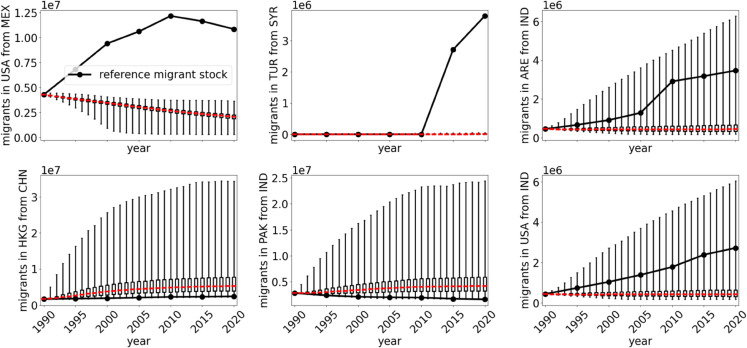
National results. Migrant stocks corresponding to the six largest errors shown in S7 Table. The red line marks the median value of the simulation while the boxes indicate the lower and upper quartile. The whiskers show the 95% prediction range.

The time series of the six largest deviations on the country level are shown in Fig 9 while a detailed description is presented in S7 Appendix.

Even though we work only with global parameters our 95% prediction range covers most of the migrant stocks in the cases of India-UAE, China-Hong Kong, India-Pakistan, and India-USA. On the other hand, we find large quantitative and qualitative disagreement in the migrant populations Mexico-USA and Syria-Türkiye where again regional parameters are key ingredients to understand the historical development. The non-linear trend of the Mexican migrant population in USA has it complex roots not only in changing immigration policies but also economic factors such as the Great Recession in 2008 [66]. To properly capture the full mechanisms of the migrant stock time evolution may even require bilateral parameterization to account for regional agreements that impact migration [67]. Similarly, our simplified global model cannot account for the civil war in Syria which led to large refugee numbers in Türkiye since 2010. Subtracting refugee numbers in the reference migrant stock significantly reduces the deviation (see S5 Figure). The model performance is further studied in S8 Appendix where we show that our model compares well to other state-of-the-art approaches given the differences in parameter numbers and target variables.

In summary, we find that our model can reproduce several trends on different aggregation levels while in certain contexts we need more regional information to account for important migration factors. Such improvements can be performed by replacing the global parameters of the model by regional parameters. Nevertheless, our migration sampling which relies on a global demographic, economic, and social parameterization has proven to be a promising starting point for further development.

## Discussion

In this work we presented a new model for international migration based on stochastic evolution equations. While the evolution equations incorporate migration flow accounting we rely on stochastic sampling in order to estimate migration rates, return rates and destination countries. We include knowledge about demographic, economic, and social determinants of migration by partitioning the migration data according to population size, GDPc ratio and relative migrant population size before extracting the migration flow distributions that are used for stochastic sampling. The probabilistic component partially acknowledges for our incomplete understanding of the migration process itself but also biases in the migration data.

We showed that the migration data partitions lead to probability distributions that are consistent with existing theories of migration. This includes the observation that median migration rates are smaller in large countries and the importance of migrant networks for the migration destination choice. In the latter case we observed changes in orders of magnitude as a function of the relative migrant population size while the effects of the GDPc ratio between origin and destination country has a much smaller effect. Moreover, we have shown that stochastic sampling is a key component of the model, as a purely deterministic version of the model leads to qualitatively different results that are inconsistent with reference data. This underlines the importance of the non-Gaussian structure of the migration data and the non-trivial time dynamics of the migration flow modeling. Despite the few global calibration parameters of our model we were able to reproduce important trends on the global, regional, and country income group level. On the national level, we showed that the ten largest differences between reference and simulated migrant stocks only exceed several orders of magnitude in the case of the Syrian migrant population in Türkiye which we can attribute to refugees from the Syrian civil war. Moreover, the observed deviations in certain world regions can most likely be attributed to migration policy and historical events which are not yet included within our model but can be flexibly incorporated within the stochastic framework. This refinement is performed by replacing the global parameters with parameters that may depend on the location and time to account for confined impacts like labor migration agreements or historical shocks. For example, an economic crisis in region X will probably reduce migration to X [68]. In terms of modeling this means that the distribution that selects the share of migration flow to X (see Eq (10) and Fig 5) will have to be shifted towards smaller probabilities. This can be done by subtracting a certain value from the mean parameter of the distribution, i.e. $\mu_{\left(g^{*},d^{*}\right)}^{s}$ in Eq (10) decreases. That amount of reduction can be calibrated to account for the severity of the crisis. On the other hand, the variance of the emigration probability distribution ($\sigma_{P^{*}}^{m}$ in Eq (4)) can be increased in X to account for higher uncertainties in migration decisions. Finally, such events may be restricted in time which can be modeled by introducing decay functions to the parameters that restore the original values of these parameters while the crisis is fading away. In terms of migration policies one can proceed in a similar way while also making use of policy databases such as the DEMIG VISA data [69], the GLOBALCIT citizenship law dataset [70] or the Migrant Integration Policy Index (MIPEX) [71]. These datasets can be used to calibrate the stochastic parameters as a function of migration-policy indicators for different regions.

Additionally, the stochastic language allows for more flexible scenario building when applied to future projections. These promising results highlight the complex nature of international migration and show that a combination of deterministic and stochastic tools can be a meaningful way to address this complexity.

## Supporting information

S1 Appendix Migration regression. (PDF)

S2 Appendix Numerical example for Eqs (1) and (2).
(PDF)

S3 Appendix Convergence. (PDF)

S4 Appendix Further information on the sampling procedure. (PDF)

S5 Appendix Further information on data. (PDF)

S6 Appendix Simulation results from the destination perspective. (PDF)

S7 Appendix National results. (PDF)

S8 Appendix (PDF)

S1 Table Emigration rates regression. Top three best-fitting probability distributions for the emigration rates and their respective sum of squared error.
(TIFF)

S2 TableEmigration rates distribution parameters.Parameters of the probability distribution for the partition $\left\{P_{1},P_{2},P_{3},P_{4}\right\}$ (Eq (3)), where *μ* denotes the location, *σ* the scale, *k* the degrees of freedom parameter, and *c* the non-centrality parameter.(TIFF)

S3 Table Emigration shares regression. Top three best-fitting probability distributions for the bilateral emigration shares and their respective sum of squared error.
(TIFF)

S4 TableEmigration shares distribution parameters.Parameters of the probability distributions for the partition G×D (Eq 5), where *μ* denotes the location, *σ* the scale, and *a*,*b* are shape parameters of Weibull maximum distribution.(TIFF)

S5 Table Return rates regression. Top three best-fitting probability distributions for the return migration rates and the respective sum of squared error.
(TIFF)

S6 Table Input data and calibration. Calibration and input data for the stochastic evolution model. In the last column we describe the adjustments performed before calibrating the model.
(TIFF)

S7 Table National results. Ten largest differences between observed and predicted migrant stocks in 2020. The first two columns contain information about the origin and residence country of the migrant population respectively. For comparison we show the observed migrant stocks next to the model median values, and the 80% prediction intervals. The absolute error is defined as the difference between model median and observed migrant stock and the relative error is given by the ratio between absolute error and observed migrant stock.
(TIFF)

S1 Fig Return rates distribution. Return rate regression result (red line) for the return migration data where the blue histogram represents the return migration rates data. The return rates are concentrated around the median value (vertical dashed line).
(TIFF)

S2 Fig Sampling convergence. Convergence of the stochastic evolution equations. (a) Convergence of the median total migrant stock as a function of the sample size. (b) Distribution of total stocks at sample size 10^5^.
(TIFF)

S3 Fig Destination region results. Migrant stocks with respect to the destination region. The red line marks the median value of the simulation while the boxes indicate the lower and upper quartile values. The whiskers mark the 95% prediction interval.
(TIFF)

S4 Fig Destination income group results. Regional migrant stocks with respect to the destination income group. The red line marks the median value of the simulation while the boxes indicate the lower and upper quartile. The whiskers show the 95% prediction range.
(TIFF)

S5 Fig National results. Same as Fig 9 but here we subtracted refugee numbers [?] from the reference migrant stocks.
(TIFF)

S6 Fig Total migrant stocks in Sweden and Japan. Total migrant stock comparison between our model and reference data for Sweden and Japan. The whiskers signify the 99% prediction interval while the box represents the 50% prediction range and the red marker is the median value.
(TIFF)
