# Supplementary material for: Stochastic evolution model for international migration
Source: PLoS One. 2025 Oct 7;20(10):e0332886. doi: 10.1371/journal.pone.0332886 (PMC12503288; doi:10.1371/journal.pone.0332886)
Supplement: S1 Appendix — (PDF) [file pone.0332886.s001.pdf]

PLOS ONE

RESEARCH ARTICLE

Stochastic evolution model for international migration

Karim Zantout[¤*], **Jacob Schewe**

Transformation Pathways Department, Potsdam Institute for Climate Impact Research, Potsdam, Brandenburg, Germany

¤ Current address: Faculty for Information Management and Media, University of Applied Sciences Karlsruhe, Karlsruhe, Baden-Württemberg, Germany
* karim.zantout@h-ka.de

Check for updates

OPEN ACCESS

Citation: Zantout K, Schewe J (2025) Stochastic evolution model for international migration. PLoS One 20(10): e0332886. https://doi.org/10.1371/journal.pone.0332886

Editor: Nicolò Pecora, Università Cattolica del Sacro Cuore Sede di Piacenza e Cremona Facoltà di Economia: Universita Cattolica del Sacro Cuore Facolta di Economia e Giurisprudenza, ITALY

Received: February 10, 2025

Accepted: September 5, 2025

Published: October 7, 2025

Copyright: © 2025 Zantout, Schewe. This is an open access article distributed under the terms of the Creative Commons Attribution License, which permits unrestricted use, distribution, and reproduction in any medium, provided the original author and source are credited.

Data availability statement: All relevant data are within the manuscript and its Supporting information files. Therefore, our submission contains our "minimal data set". In addition, we provide the final results to reproduce the graphs in the Supporting information.

Funding: The research was financially supported through the European Union Horizon 2020 programme (HABITABLE project (Grant 869395)) and the German Federal Foreign Office

Abstract

We present a new international migration model that combines stochastic sampling techniques with dynamic accounting of flows by means of evolution equations. Migration flows are sampled from paramaterized probability distributions based on reported migration flow data that is partitioned by socio-economic covariates. This method allows for non-trivial time evolution that goes beyond extrapolation, while requiring minimal prior knowledge about the elusive processes driving migration flows. It thus combines the advantages of different existing modeling approaches. In hindcasts our model compares well with bilateral migrant stock data in many world regions and country income groups. Moreover, we observe a significant difference between the full model and its deterministic formulation, which highlights the non-Gaussian and interdependent nature of migration flow distributions and corroborates the use of a stochastic dynamic approach. Our model can be flexibly extended with additional information, e.g. regional migration policies, which are expected to further improve the agreement with data.

Supporting information

S1 Appendix. Migration regression. Having labeled the emigration rates data based on the partition in the definitions (3) we proceed by finding the best-fitting probability distribution function. This is done by testing all available continuous distribution functions within the scipy library [72] and summing the sum of squared errors (SSE) from each data set using the maximum likelihood estimation. The results are shown in S1 Table. The best fit is given by the Jones and Faddy skew-t distribution which leads to probability parameters given in S2 Table. Following the same procedure as for the emigration rate calibration, we find Weibull maximum distribution to yield the best fit to the bilateral emigration share data as shown in S3 Table. The resulting probability distribution parameters are given in S4 Table, where we dropped all combinations of GDPc ratio and relative migrant population size without sufficient data point to converge the regression procedure.
In the case of return migration rates we find Student's t distribution to be the best-fitting probability distribution (see S1 Figure and S5 Table).

The resulting parameters are given by

$$\nu^r = 1.71, \ \mu^r = -1.64, \ \sigma^r = 7.97 \cdot 10^{-2}, \tag{15}$$

where ν^r is the degree of freedom of the distribution, μ^r is the location of the distribution, σ^r is the scale.

References

1. Brouwer J, van der Woude M, van der Leun J. Framing migration and the process of crimmigration: A systematic analysis of the media representation of unauthorized immigrants in the Netherlands. Eur J Criminol. 2017;14(1):100–19. https://doi.org/10.1177/1477370816640136 PMID: 28596711
2. Vezovnik A. Securitizing Migration in Slovenia: A Discourse Analysis of the Slovenian Refugee Situation. Journal of Immigrant & Refugee Studies. 2017;16(1–2):39–56. https://doi.org/10.1080/15562948.2017.1282576
3. Cottier F, Flahaux M-L, Ribot J, Seager R, Ssekajja G. Framing the frame: Cause and effect in climate-related migration. World Development. 2022;158:106016. https://doi.org/10.1016/j.worlddev.2022.106016
4. Bijak J. Forecasting Migration: Selected Models and Methods. The Springer Series on Demographic Methods and Population Analysis. Springer Netherlands. 2010. p. 53–87. https://doi.org/10.1007/978-90-481-8897-0_4
5. Castles S, Miller MJ. The age of migration. Basingstoke: Macmillan. 2009.
6. de Haas H. A theory of migration: the aspirations-capabilities framework. Comp Migr Stud. 2021;9(1):8. https://doi.org/10.1186/s40878-020-00210-4 PMID: 33680858
7. Rees PH. Multistate Demographic Accounts: Measurement and Estimation Procedures. Environ Plan A. 1980;12(5):499–531. https://doi.org/10.1068/a120499
8. Massey DS. Social structure, household strategies, and the cumulative causation of migration. Popul Index. 1990;56(1):3–26. PMID: 12316385
9. Kritz MM, Lim LL, Zlotnik H. International migration systems: a global approach. Clarendon Press. 1992.
10. Oberg S, Wils AB. East-West migration in Europe: can migration theories help estimate the numbers? Popnet. 1992;(22):1–7. PMID: 12286068
11. Zelinsky W. The hypothesis of the mobility transition. Geographical Review. 1971;61(2):219–49.
12. Bencek D, Schneiderheinze C. Higher economic growth in poor countries, lower migration flows to the OECD: revisiting the migration hump with panel data. Kiel Institute for the World Economy (IfW Kiel). 2020. https://ideas.repec.org/p/zbw/ifwkwp/2145.html
13. Clemens MA. Migration on the Rise, a Paradigm in Decline: The Last Half-Century of Global Mobility. AEA Papers and Proceedings. 2022;112:257–61. https://doi.org/10.1257/pandp.20221050
14. Kupiszewska D, Nowok B. Comparability of Statistics on International Migration Flows in the European Union. Comparability of Statistics on International Migration Flows in the European Union. John Wiley & Sons, Ltd. 2007. p. 41–71.
15. Buettner T. Stocktaking of Migration Data. 42. KNOMAD. 2022.
16. Abel GJ, Cohen JE. Bilateral international migration flow estimates for 200 countries. Sci Data. 2019;6(1):82. https://doi.org/10.1038/s41597-019-0089-3 PMID: 31209218
17. Massey DS, Arango J, Hugo G, Kouaouci A, Pellegrino A, Taylor JE. Theories of International Migration: A Review and Appraisal. Population and Development Review. 1993;19(3):431–66.
18. Piguet E. Theories of voluntary and forced migration. Routledge handbook of environmental displacement and migration. Routledge. 2018. p. 17–28.
19. de Sherbinin A, Grace K, McDermid S, van der Geest K, Puma MJ, Bell A. Migration Theory in Climate Mobility Research. Front Clim. 2022;4. https://doi.org/10.3389/fclim.2022.882343
20. Stewart JQ. An inverse distance variation for certain social influences. Science. 1941;93(2404):89–90. https://doi.org/10.1126/science.93.2404.89 PMID: 17729640
21. Zipf GK. The P1 P2/D Hypothesis: On the Intercity Movement of Persons. American Sociological Review. 1946;11(6):677–86.
22. Lowry IS. Migration and metropolitan growth: two analytical models. Los Angeles, Calif.: Chandler Publishing Company. 1966.
23. Vanderkamp J. The gravity model and migration behaviour: an economic interpretation. Journal of Economic Studies. 1977;4(2):89–102. https://doi.org/10.1108/eb002472

24. Anderson JE. The Gravity Model. Annu Rev Econ. 2011;3(1):133–60. https://doi.org/10.1146/annurev-economics-111809-125114
25. Beine M, Bertoli S, Fernández-Huertas Moraga J. A Practitioners' Guide to Gravity Models of International Migration. World Economy. 2015;39(4):496–512. https://doi.org/10.1111/twec.12265
26. Rogers A. Introduction to multiregional mathematical demography. Hoboken, NJ: Wiley. 1975.
27. Rogers A. Introduction to Multistate Mathematical Demography. Environ Plan A. 1980;12(5):489–98. https://doi.org/10.1068/a120489
28. Kupiszewski M, Kupiszewska D. MULTIPOLES: A Revised Multiregional Model for Improved Capture of International Migration. Population Dynamics and Projection Methods. Springer Netherlands. 2011. p. 41–60. https://doi.org/10.1007/978-90-481-8930-4_3
29. Desmet K, Nagy DK, Rossi-Hansberg E. The Geography of Development. Journal of Political Economy. 2018;126(3):903–83. https://doi.org/10.1086/697084
30. Docquier F. Long-Term Trends in International Migration: Lessons from Macroeconomic Model. Economics and Business Review. 2018;4(1):3–15. https://doi.org/10.18559/ebr.2018.1.1
31. Dao TH, Docquier F, Maurel M, Schaus P. Global migration in the twentieth and twenty-first centuries: the unstoppable force of demography. Review of World Economics. 2021;157(2):417–49. https://doi.org/10.1007/s10290-020-00402-1
32. Beyer RM, Schewe J, Lotze-Campen H. Gravity models do not explain, and cannot predict, international migration dynamics. Humanities and Social Sciences Communications. 2022;9(1). https://doi.org/10.1057/s41599-022-01067-x
33. Cohen JE, Roig M, Reuman DC, GoGwilt C. International migration beyond gravity: a statistical model for use in population projections. Proceedings of the National Academy of Sciences. 2008;105(40):15269–74. https://doi.org/10.1073/pnas.0808185105 PMID: 18824693
34. Kim K, Cohen JE. Determinants of International Migration Flows to and from Industrialized Countries: A Panel Data Approach beyond Gravity. International Migration Review. 2010;44(4):899–932. https://doi.org/10.1111/j.1747-7379.2010.00830.x
35. Goodman LA. Statistical Methods for the Mover-Stayer Model. Journal of the American Statistical Association. 1961;56(296):841–68.
36. Rogers A. A Markovian policy model of interregional migration. Papers of the Regional Science Association. 1966;17(1):205–24. https://doi.org/10.1007/bf01982518
37. Azose JJ, Raftery AE. Bayesian Probabilistic Projection of International Migration. Demography. 2015;52(5):1627–50. https://doi.org/10.1007/s13524-015-0415-0 PMID: 26358699
38. Azose JJ, Ševčíková H, Raftery AE. Probabilistic population projections with migration uncertainty. Proceedings of the National Academy of Sciences. 2016;113(23):6460–5. https://doi.org/10.1073/pnas.1606119113 PMID: 27217571
39. Welch NG, Raftery AE. Probabilistic forecasts of international bilateral migration flows. Proceedings of the National Academy of Sciences. 2022;119(35):e2203822119. https://doi.org/10.1073/pnas.2203822119 PMID: 35994637
40. Raymer J, Wiśniowski A, Forster JJ, Smith PWF, Bijak J. Integrated Modeling of European Migration. Journal of the American Statistical Association. 2013;108(503):801–19. https://doi.org/10.1080/01621459.2013.789435
41. Weidlich W, Andersson AE, Haag G, Haag G, Holmberg I, Ledent J. Interregional migration: dynamic theory and comparative analysis. Springer Berlin Heidelberg. 2012.
42. UN DESA. International migrant stock 2020. UN DESA. 2022. https://www.un.org/development/desa/pd/content/international-migrant-stock
43. Kulu H. Migration and Fertility: Competing Hypotheses Re-examined. Eur J Population. 2005;21(1):51–87. https://doi.org/10.1007/s10680-005-3581-8
44. Stirbu I, Kunst AE, Vlems FA, Visser O, Bos V, Deville W, et al. Cancer mortality rates among first and second generation migrants in the Netherlands: Convergence toward the rates of the native Dutch population. Int J Cancer. 2006;119(11):2665–72. https://doi.org/10.1002/ijc.22200 PMID: 16929492
45. Beine M, Docquier F, Schiff M. International migration, transfer of norms and home country fertility. The Canadian Journal of Economics/ Revue canadienne d'Economique. 2013;46(4):1406–30.
46. Mussino E, Van Raalte AA. Immigrant Fertility: A Comparative Study between Italy and Russia. International Migration. 2012;51(2):148–64. https://doi.org/10.1111/j.1468-2435.2012.00760.x
47. Aldridge RW, Nellums LB, Bartlett S, Barr AL, Patel P, Burns R, et al. Global patterns of mortality in international migrants: a systematic review and meta-analysis. Lancet. 2018;392(10164):2553–66. https://doi.org/10.1016/S0140-6736(18)32781-8 PMID: 30528484
48. Impicciatore R, Gabrielli G, Paterno A. Migrants' Fertility in Italy: A Comparison Between Origin and Destination. Eur J Popul. 2020;36(4):799–825. https://doi.org/10.1007/s10680-019-09553-w PMID:

32999641

49. Simini F, González MC, Maritan A, Barabási A-L. A universal model for mobility and migration patterns. Nature. 2012;484(7392):96–100. https://doi.org/10.1038/nature10856 PMID: 22367540
50. Taylor JE. Differential migration, networks, information and risk. Migration, Human Capital and Development. 1986. p. 147–71.
51. Faist T. The Volume and Dynamics of International Migration and Transnational Social Spaces. Oxford University Press. 2000. https://doi.org/10.1093/acprof:oso/9780198293910.001.0001
52. Beine M, Docquier F, Ozden C. Diaspora effects in international migration: key questions and methodological issues. 10–14. Department of Economics at the University of Luxembourg. 2010.
53. Pries L. New transnational social spaces: International migration and transnational companies in the early twenty-first century. Routledge Research in Transnationalism: Taylor & Francis. 2013.
54. Dyrting S. Smoothing migration intensities with P-TOPALS. DemRes. 2020;43:1607–50. https://doi.org/10.4054/demres.2020.43.55
55. Rees PH. The Measurement of Migration, from Census Data and other Sources. Environ Plan A. 1977;9(3):247–72. https://doi.org/10.1068/a090247
56. Abel GJ, Cohen JE. Bilateral international migration flow estimates updated and refined by sex. Sci Data. 2022;9(1):173. https://doi.org/10.1038/s41597-022-01271-z PMID: 35422105
57. Rogerson PA. Migration analysis using data with time intervals of differing widths. Papers in Regional Science. 1990;68(1):97–106. https://doi.org/10.1111/j.1435-5597.1990.tb01196.x
58. Rogers A, Raymer J, Newbold KB. Reconciling and translating migration data collected over time intervals of differing widths. The Annals of Regional Science. 2003;37(4):581–601. https://doi.org/10.1007/s00168-003-0128-y
59. UN DESA. World Population Prospects 2024: Methodology of the United Nations Population Estimates and Projections. 2024.
60. World Bank. 2022. https://data.worldbank.org/indicator/SP.POP.TOTL
61. Jordahl K, den Bossche JV, Fleischmann M, Wasserman J, McBride J, Gerard J. Geopandas/geopandas: v0.8.1. 2020.
62. Korobkov AV, Zaionchkovskaia ZA. The changes in the migration patterns in the post-Soviet states: the first decade. Communist and Post-Communist Studies. 2004;37(4):481–508. https://doi.org/10.1016/j.postcomstud.2004.09.004
63. Weinar A. A Look at Migrations in the Post-Soviet Space – the Case of Eastern Europe, South Caucasus and Russian Federation. International Migration. 2014;52(5):47–51. https://doi.org/10.1111/imig.12168
64. Flahaux M-L, De Haas H. African migration: trends, patterns, drivers. CMS. 2016;4(1). https://doi.org/10.1186/s40878-015-0015-6
65. UNHCR. Global Trends Report 2022. 2022. https://www.unhcr.org/global-trends-report-2022
66. Gutiérrez RA. Mexican immigration to the United States. 2019. https://oxfordre.com/americanhistory/view/10.1093/acrefore/9780199329175.001.0001/acrefore-9780199329175-e-146
67. Elsasser AA. Migration from Mexico to the US: the impacts of NAFTA on Mexico and the United States and what to do going forward. International Review of Business and Economics. 2018;2(1):2.
68. Green T, Winters LA. Economic Crises and Migration: Learning from the Past and the Present. The World Economy. 2010;33(9):1053–72. https://doi.org/10.1111/j.1467-9701.2010.01313.x
69. DEMIG. DEMIG VISA version 1.4. Oxford: International Migration Institute, University of Oxford. 2022. https://www.migrationinstitute.org/data/demig-data/demig-visa-data
70. Vink M, Tegunimataka A, Peters F, Bevelander P. Long-Term Heterogeneity in Immigrant Naturalization: The Conditional Relevance of Civic Integration and Dual Citizenship. European Sociological Review. 2021;37(5):751–65. https://doi.org/10.1093/esr/jcaa068
71. Solano G, Huddleston T. Migrant integration policy index 2020. 2020.
72. Virtanen P, Gommers R, Oliphant TE, Haberland M, Reddy T, Cournapeau D, et al. SciPy 1.0: fundamental algorithms for scientific computing in Python. Nat Methods. 2020;17(3):261–72. https://doi.org/10.1038/s41592-019-0686-2 PMID: 32015543
73. Van Rossum G, Drake FL. Python 3 Reference Manual. Scotts Valley, CA: CreateSpace. 2009.
74. de Haas H, Natter K, Vezzoli S. Conceptualizing and measuring migration policy change. Comparative Migration Studies. 2015;3(1):15. https://doi.org/10.1186/s40878-015-0016-5 PMID: 32337161
75. de Haas H, Natter K, Vezzoli S. Growing Restrictiveness or Changing Selection? The Nature and Evolution of Migration Policies1. International Migration Review. 2018;52(2):324–67. https://doi.org/10.1111/imre.12288

76. UNHCR. UNHCR Refugee Population Statistics Database. 2024. https://www.unhcr.org/refugee-statistics

77. Kluge L, Bucaro OO, K C S, Yildiz D, Abel G, Schewe J. A multidimensional global migration model for use in cohort-component population projections. Demographic Research. 2024;51:323–76.
