# Supplementary figures and images for: Stochastic evolution model for international migration
Source: PLoS One. 2025 Oct 7;20(10):e0332886. doi: 10.1371/journal.pone.0332886 (PMC12503288; doi:10.1371/journal.pone.0332886)

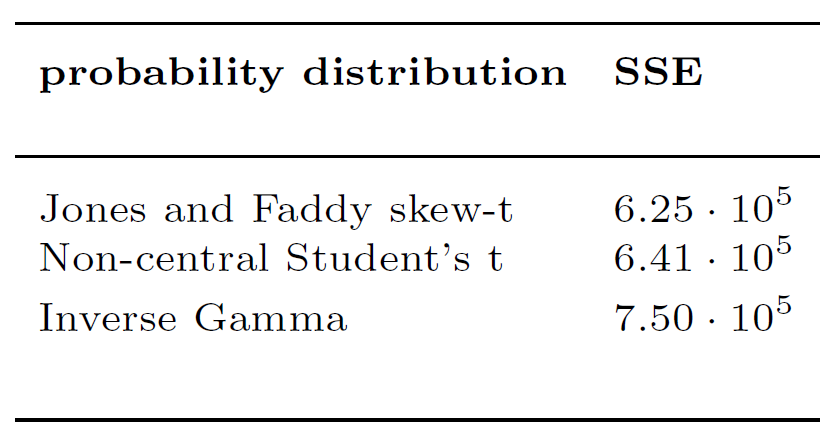

Supplement: S1 Table — Top three best-fitting probability distributions for the emigration rates and their respective sum of squared error. (TIFF) [file pone.0332886.s009.tif]

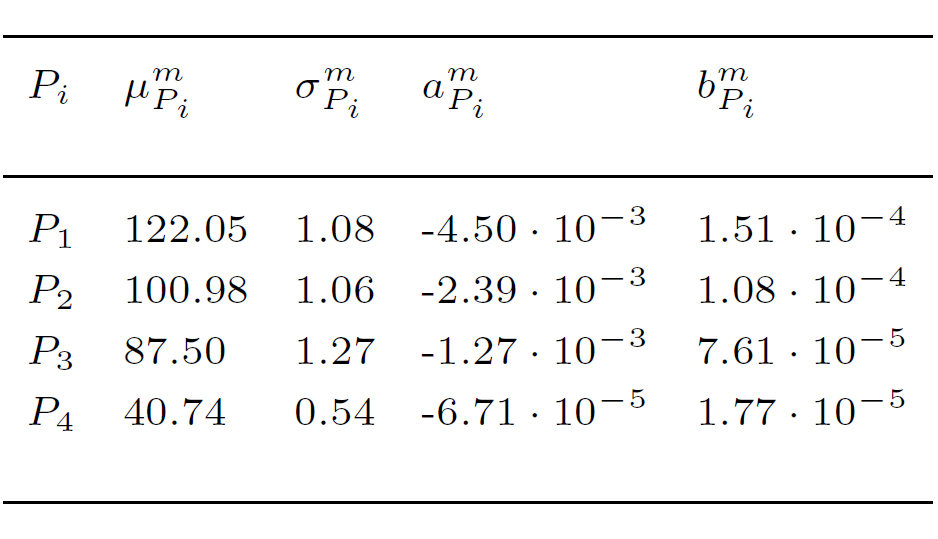

Supplement: S2 Table — Parameters of the probability distribution for the partition {P1,P2,P3,P4} (Eq (3)), where μ denotes the location, σ the scale, k the degrees of freedom parameter, and c the non-centrality parameter. (TIFF) [file pone.0332886.s010.tif]

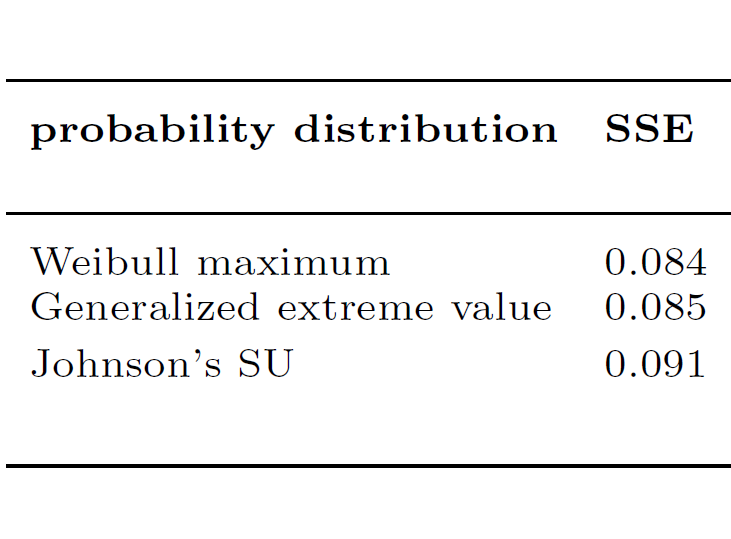

Supplement: S3 Table — Top three best-fitting probability distributions for the bilateral emigration shares and their respective sum of squared error. (TIFF) [file pone.0332886.s011.tif]

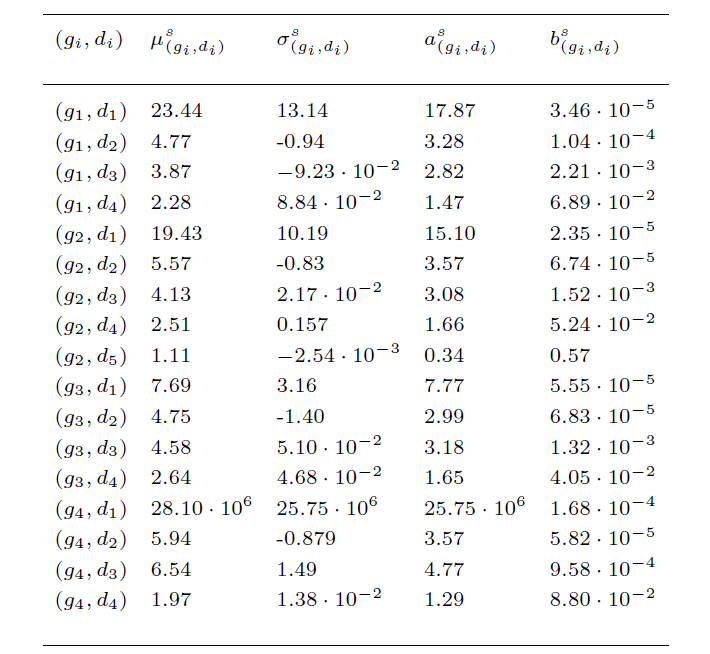

Supplement: S4 Table — Parameters of the probability distributions for the partition G×D (Eq 5), where μ denotes the location, σ the scale, and a,b are shape parameters of Weibull maximum distribution. (TIFF) [file pone.0332886.s012.tif]

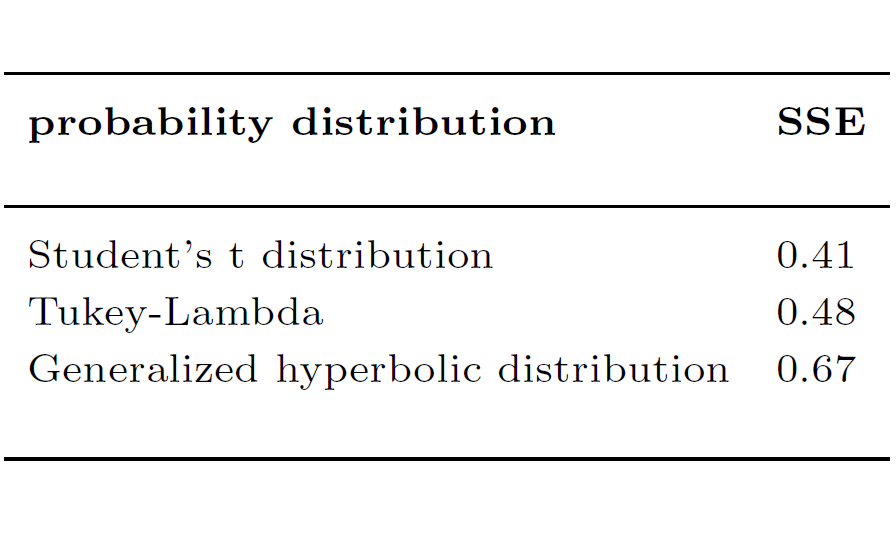

Supplement: S5 Table — Top three best-fitting probability distributions for the return migration rates and the respective sum of squared error. (TIFF) [file pone.0332886.s013.tif]

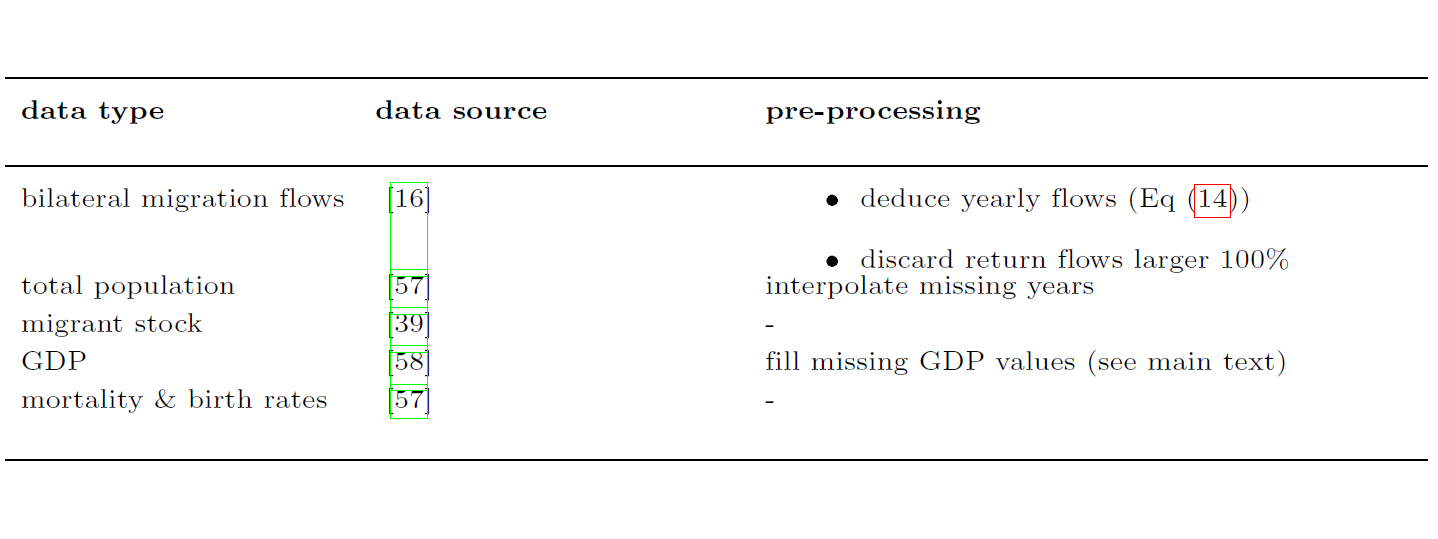

Supplement: S6 Table — Calibration and input data for the stochastic evolution model. In the last column we describe the adjustments performed before calibrating the model. (TIFF) [file pone.0332886.s014.tif]

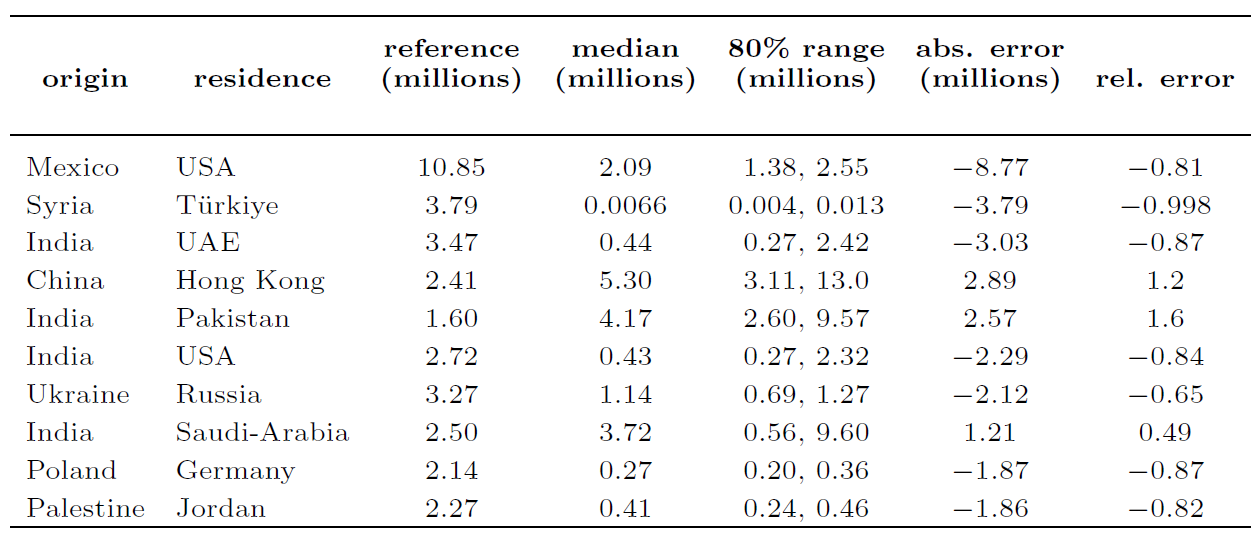

Supplement: S7 Table — Ten largest differences between observed and predicted migrant stocks in 2020. The first two columns contain information about the origin and residence country of the migrant population respectively. For comparison we show the observed migrant stocks next to the model median values, and the 80% prediction intervals. The absolute error is defined as the difference between model median and observed migrant stock and the relative error is given by the ratio between absolute error and observed migrant stock. (TIFF) [file pone.0332886.s015.tif]

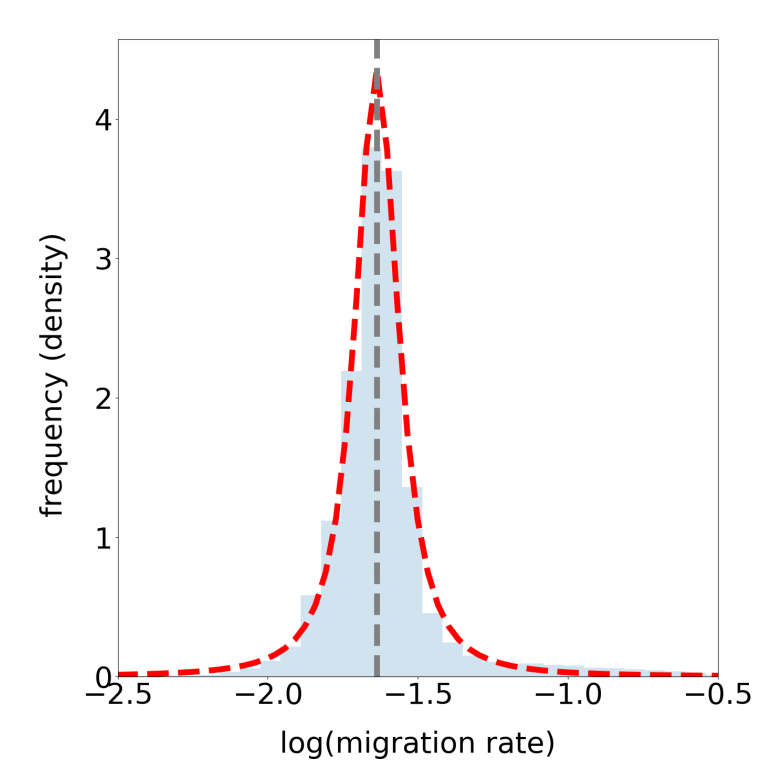

Supplement: S1 Fig — Return rate regression result (red line) for the return migration data where the blue histogram represents the return migration rates data. The return rates are concentrated around the median value (vertical dashed line). (TIFF) [file pone.0332886.s016.tif]

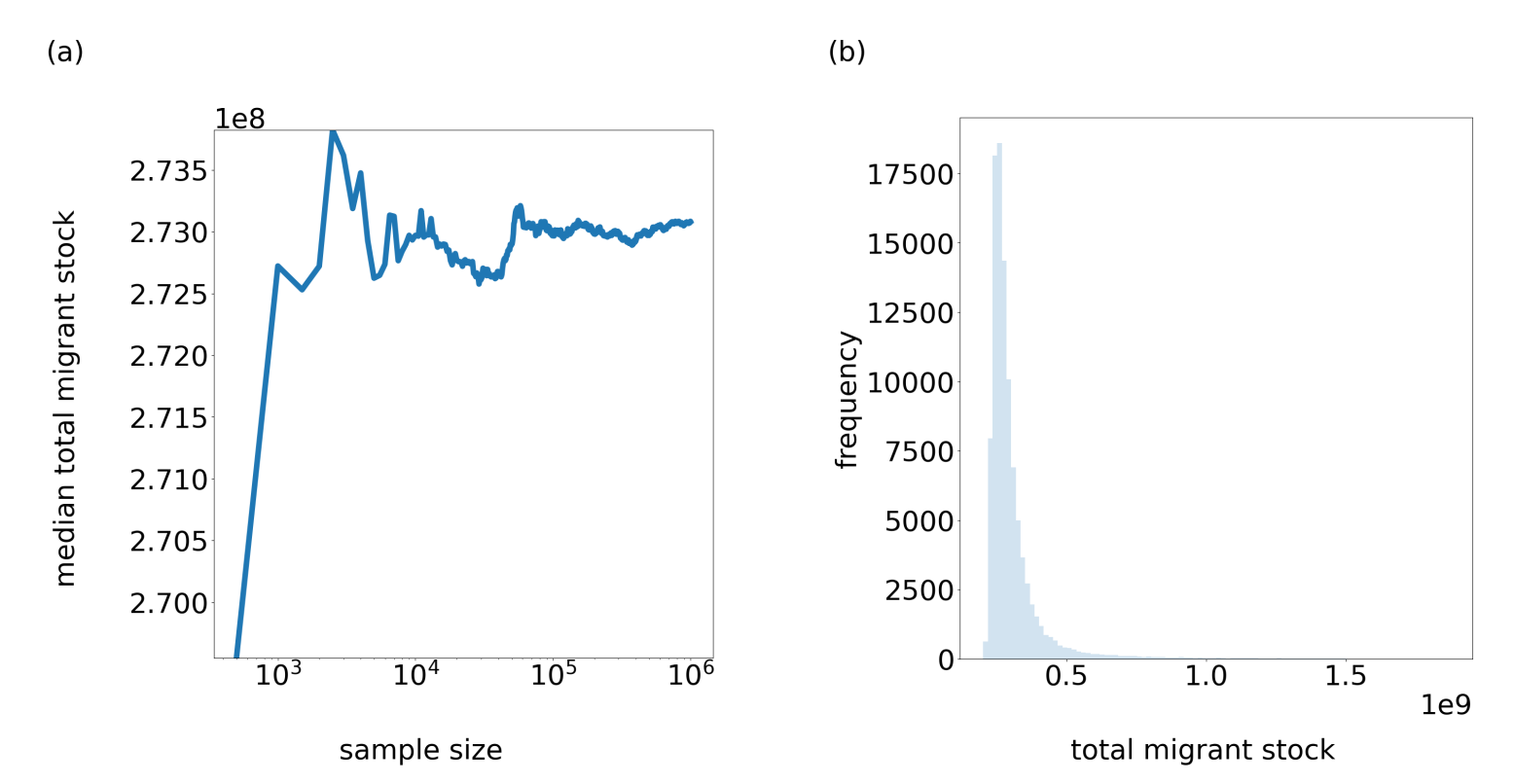

Supplement: S2 Fig — Convergence of the stochastic evolution equations. (a) Convergence of the median total migrant stock as a function of the sample size. (b) Distribution of total stocks at sample size 105. (TIFF) [file pone.0332886.s017.tif]

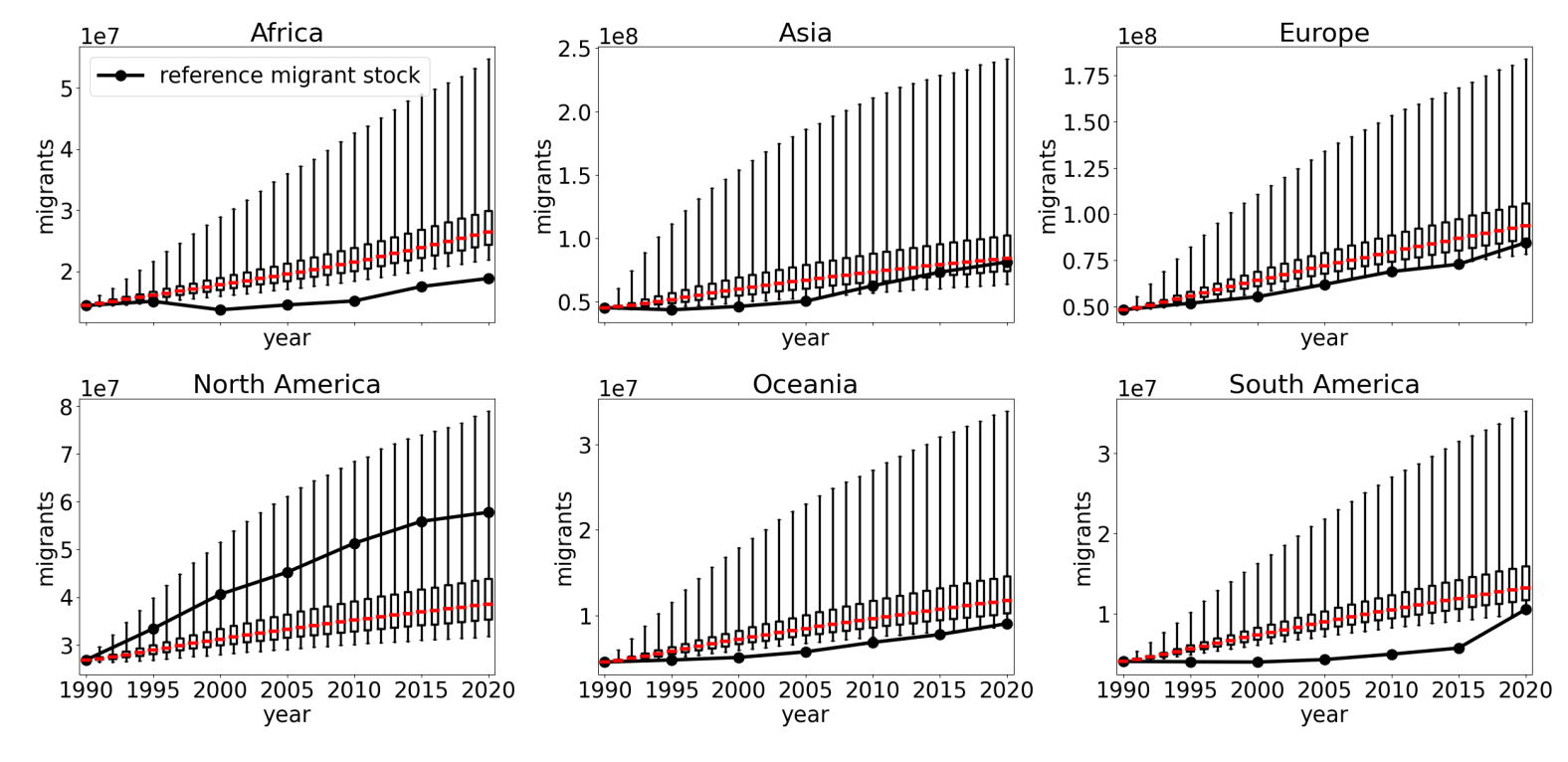

Supplement: S3 Fig — Migrant stocks with respect to the destination region. The red line marks the median value of the simulation while the boxes indicate the lower and upper quartile values. The whiskers mark the 95% prediction interval. (TIFF) [file pone.0332886.s018.tif]

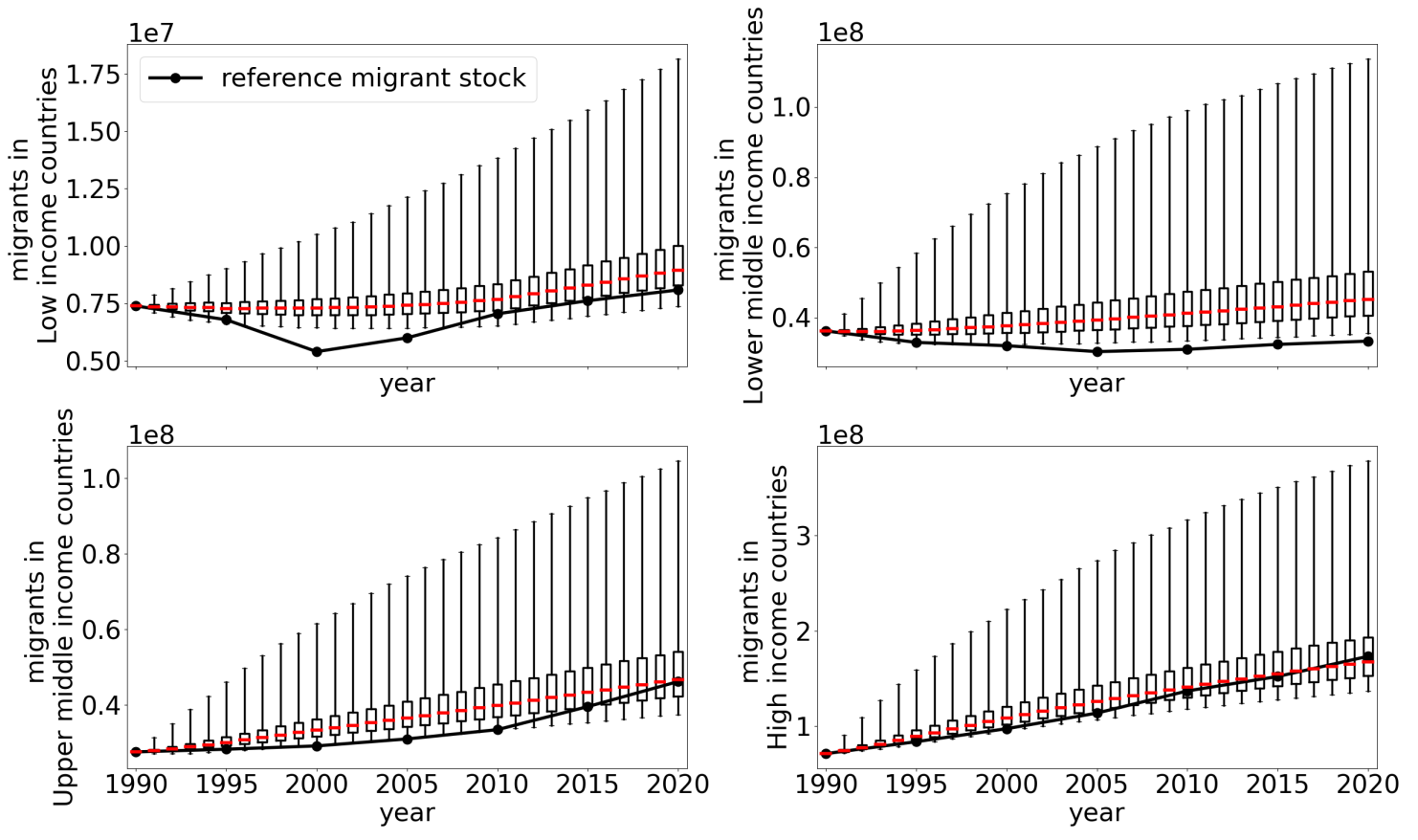

Supplement: S4 Fig — Regional migrant stocks with respect to the destination income group. The red line marks the median value of the simulation while the boxes indicate the lower and upper quartile. The whiskers show the 95% prediction range. (TIFF) [file pone.0332886.s019.tif]

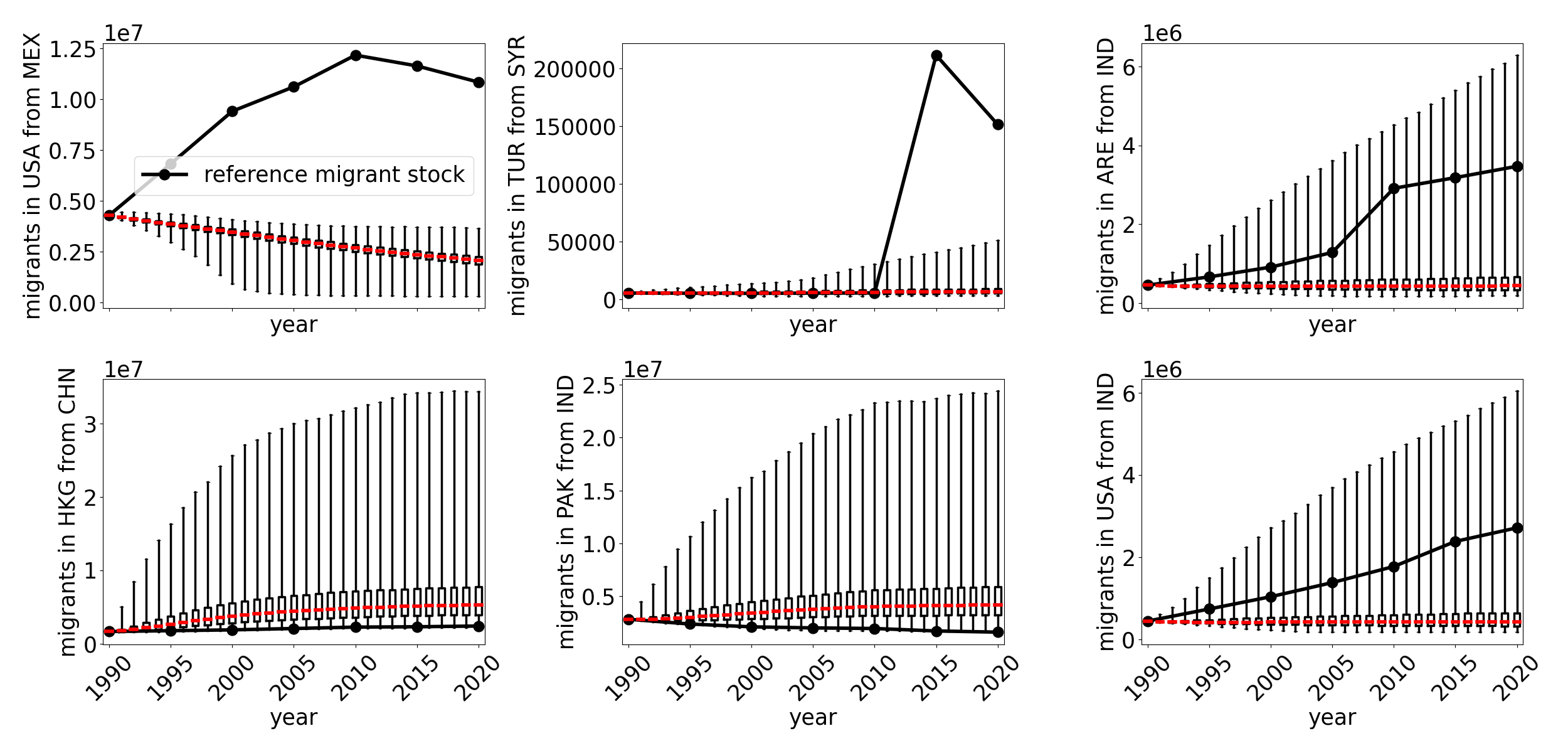

Supplement: S5 Fig — Same as Fig 9 but here we subtracted refugee numbers [?] from the reference migrant stocks. (TIFF) [file pone.0332886.s020.tif]

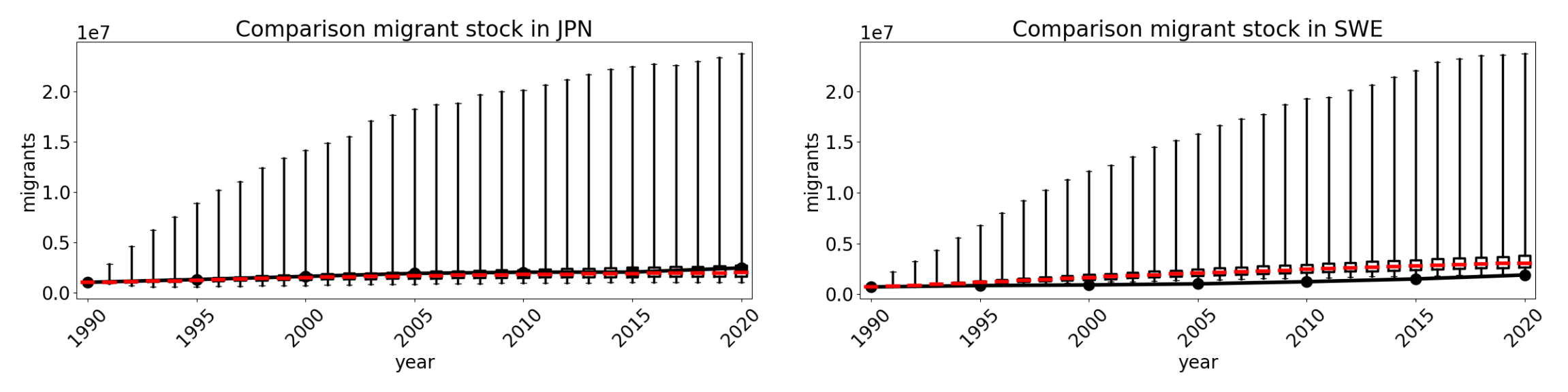

Supplement: S6 Fig — Total migrant stock comparison between our model and reference data for Sweden and Japan. The whiskers signify the 99% prediction interval while the box represents the 50% prediction range and the red marker is the median value. (TIFF) [file pone.0332886.s021.tif]
